# Airway Remodelling in Asthma and COPD: Findings, Similarities, and Differences Using Quantitative CT

**DOI:** 10.1155/2012/670414

**Published:** 2012-02-16

**Authors:** Gaël Dournes, François Laurent

**Affiliations:** ^1^Department of Thoracic and Cardiovascular Imaging, CHU Bordeaux, avenue de Magellan, 33604 Pessac, France; ^2^Laboratory of Cellular Respiratory Physiology, Centre de Recherche Cardio-Thoracique de Bordeaux, INSERM U1045. University Bordeaux Segalen, 146, rue Léo Saignat, 33076 Bordeaux, France

## Abstract

Airway remodelling is a well-established feature in asthma and chronic obstructive lung disease (COPD), secondary to chronic airway inflammation. The structural changes found on pathological examination of remodelled airway wall have been shown to display similarities but also differences. Computed tomography (CT) is today a remarkable tool to assess airway wall morphology *in vivo* since submillimetric acquisitions over the whole lung volume could be obtained allowing 3D evaluation. Recently, CT-derived indices extracted from CT images have been described and are thought to assess airway remodelling. This may help understand the complex mechanism underlying the remodelling process, which is still not fully understood. This paper summarizes the various methods described to quantify airway remodelling in asthma and COPD using CT, and similarities and differences between both diseases will be emphasized.

## 1. Introduction

Asthma and COPD are the most prominent obstructive lung diseases and affect millions of people with an increasing incidence. In their purest form, both clinical presentations are different [1]. Asthma is a youthful onset in nonsmokers, related with episodic and reversible airway obstruction in response to a stimulus. Conversely COPD is characterized by tobacco-related airflow obstruction, which is progressive and poorly reversible [2]. Airway remodelling is a well-established structural feature observed in both diseases, though to be the consequence of chronic airway inflammation. The complex mechanism underlying this process is not fully understood [3]. Pathologically, airway remodelling consists in structural changes within the airway wall, such as an increased epithelium basal membranous thickness, hypertrophy of the smooth muscle cell, and peribronchial fibrosis. Despite similarities, the remodelling features found in both diseases are different [4]. The epithelium appears to be more fragile in asthma, and the epithelial membrane thickness and the bronchial smooth muscle are thicker than in COPD. In severe cortico-dependent asthma, the bronchial wall remodelling involves a neoangiogenesis process. In COPD, the epithelium displays mucous metaplasia, and inflammation is associated with loss of alveolar attachments, surrounded by peribronchial fibrosis. Conversely, emphysema is the hallmark of severe COPD, which involves also destruction of alveolar walls. Computed tomography (CT) appears to be an effective and sensitive noninvasive tool to investigate morphological changes of the lung and bronchi *in vivo *[5, 6]. The whole volume of the lung can be evaluated by submillimetric acquisition allowing 3D reconstructions, and fully automatic quantification measurements are achievable using dedicated software. CT-derived indices have been defined and proposed as useful tool for evaluating airway wall remodelling [7, 8]. This literature review summarizes the methods developed to assess airway remodelling, their findings, and discuss the similarities or differences found between asthma and COPD using CT.

## 2. Quantitative CT of Large-to-Intermediate Airways

### 2.1. Quantitative CT Applied to Airway Wall

#### 2.1.1. Quantification of Airway Wall Thickness

Airway wall thickness is a nonspecific feature and a subjective visual finding increased in most obstructive lung diseases. CT methods have been developed to measure and improve the reproducibility of this assessment. Pathologically, airway wall thickness can be related to structural changes of remodelling but also to oedema and infiltration of inflammatory cells [3]. Therefore, before CT scanning, a sufficient anti-inflammatory treatment is recommended when the objective is to assess structural changes reflecting remodelling features [9, 10]. 

Conductive airways larger than 1mm in diameter are clearly visible on CT scans, and CT-derived methods for measurement of their wall dimensions have been developed. The rationale is to segment the airway intraluminal area (LA) and the total bronchial area (WT). Then the wall area (WA) corresponds to the difference WA = WT – LA. WA% represents WA normalised on WT, that is, WA% = (WA/WT) × 100. WA and LA are not independent from body height, and they should be normalised on body surface area (BSA), to reduce interindividual variability. 

**Figure 1:**
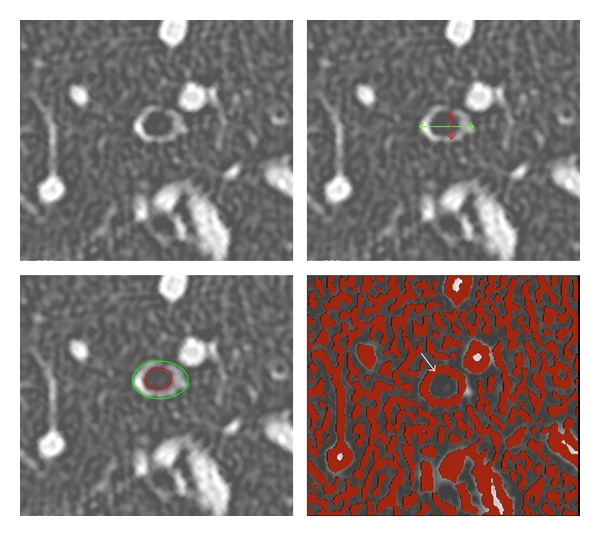
Top left. Thin section CT image, perpendicular to the fourth generation of the right apical bronchus from a random patient. Top right. Sample of manual airway wall thickness extraction. Green doublehead arrow indicates external diameter D, and red doublehead arrow shows internal diameter L. Bottom left. Sample of manual delineation of the external layer (green line) and internal layer (red line), using the mouse on the CT scan image seen in top left. Bottom right. Automatic quantification of airway wall dimension (white arrow) using a Laplacian-of-Gaussian algorithm.

Various methods have been reported to extract these data from CT images. A manual method has been first described and consists in tracing the one-dimensional internal (L) and external (D) bronchial wall (Figure 1(b)). After D and L measurements, the wall thickness indices are calculated with the assumption that the bronchial external and internal perimeters are perfectly round shaped, and the bronchial wall thickness is constant around the cross-section [11–13]. However, bronchial contours may present irregularities, especially on diseased bronchi, and the remodelling process is not circumferentially homogeneous all around the bronchial wall. Therefore, the manual method may yield measurement biases. 

To take into account the shape variability of bronchial walls, authors have proposed tracing manually on CT images the whole external and internal wall perimeters [14]. An extrapolation should be performed when the external wall abuts a vessel (Figure 1(c)). Owing to its geometry nearly perpendicular to the axial plane, the right apical bronchus (RB1) has been the most frequently studied [15]. Nevertheless, manual methods are time consuming, and exposed to intra- and interobserver variability and prone to parallax errors when the reconstructed plane is not strictly perpendicular to the measured bronchi. 

**Figure 2:**
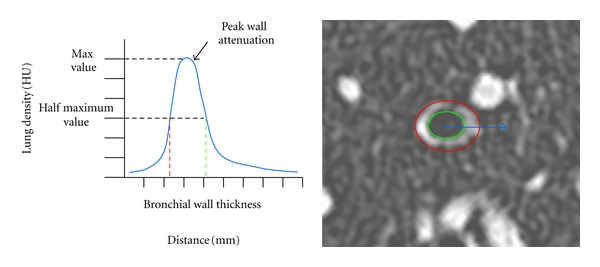
Right. Thin-section CT image perpendicular to the third generation of the right segmental apical bronchus, from a random patient. Red line indicates the external wall contour, and green line the internal layer. Left. Theoric single intensity curve (blue line) representing voxel attenuation variation along the blue arrow seen in right image.

Semiautomatic computational methods have been later developed to allow more reliable and reproducible quantification. Computational algorithms have been implemented to segment the airway wall contours and calculate airway wall dimensions [16]. The mostly employed have been the full-width-at-half-maximum (FWHM) method (Figure 2). The principle is given by the difference between the two extreme values at which the wall attenuation is equal to half to its maximum [17, 18]. Other methods have been proposed, and they all tend to minimize intra- and interobserver variability of wall thickness measurements. Though they have proven not to be interchangeable in longitudinal studies, there is still no consensus about which one is the best suitable to be used.

#### 2.1.2. Quantification of Airway Wall Attenuation

Airway wall density is the result of the X-Ray attenuation by the bronchial wall components. The information provided differs from the wall thickness since it is mainly related to the tissular components present into the airway wall. For instance a thin calcified bronchus may have a higher attenuation than a thick noncalcified other one. The use of this index has been recently emphasized. 

In a murine model of asthma, Lederlin et al. [19] studied the peribronchial attenuation (PBA) using micro CT with a spatial resolution of 46 microns. They described a manual method to segment the peribronchial space and measure the wall attenuation within a circumferential region of interest arbitrary equal to the radius of the target bronchi. In COPD patients, Washko et al. [17] and Yamashiro et al. [18] studied the peak wall attenuation (PWA) value. To understand this new biomarker, its value is extracted from bronchial wall single intensity curves, radiating outward from the centroid of the airway lumen (Figure 2). This new approach tends to better characterize the airway wall components through their global attenuation on CT images. However, both manual and semiautomatic methods using the FWHM principle are not completely independent from airway wall thickness [18].

#### 2.1.3. Bronchial Tree Segmentation

The bronchial tree can be extracted from CT acquisitions using dedicated software [20, 21]. The seeded region-growing algorithm (Figure 2) consists in a segmentation of the bronchial lumen using a bithresholding. A point is placed manually within the trachea, and voxels connected to the seed point are recruited. The skeleton of the bronchial volume is then computed, allowing perpendicular planes across the targeted bronchi to be acquired. WA indices are automatically extracted. The three-dimensional geometry of the bronchial tree can be visualised, and this is relevant knowing the heterogeneity of alterations in asthma. However, the bronchial human tree displays a mean of 24 divisions including the trachea, and only 10 divisions are reasonably achievable using this method. In COPD, the presence of emphysema areas doesn't allow the seeded region-growing algorithm to discriminate airway lumen from lung parenchyma beyond the segmental or lobar level [22].

### 2.2. Airway Wall Remodelling in Asthma and COPD Using Quantitative CT

In asthma, the remodelling process involves the whole bronchial tree, from large to small conductive airways [4]. From *in vivo* biopsies, the bronchial wall thickness, measured using CT, have been shown to correlate with remodelling features on pathological examinations. Strong correlations have been found between the WA/LA indices and the epithelial and lamina reticularis thickness [23], the smooth muscle area, and the infiltration of the smooth muscle by mast cells [24]. Correlations of increased wall thickness with function tests have also been studied, but the results are controversial. For example, since increased smooth muscle cell layer is a condition to develop airway hyper reactivity (AHR) in asthma, correlations between AHR and increased wall thickness of bronchi would be expected. However, using WA% indices, either positive [25] or negative correlations [26] have been described with AHR. Therefore, another theory has emerged, indicating that remodelled and thickened asthmatic airways are less distensible and may explain chronic airway obstruction [26]. Considering intraluminal area measurements, LA indices may indicate either bronchial dilation [27], or bronchial narrowing [24, 28], or no difference [23, 29] in asthmatic subjects when compared with control. These apparently conflicting results have been attributed to the known heterogeneity of bronchial involvement in asthma. Very few data are available so far concerning airway wall attenuation. In a murine model of asthma, the peribronchial attenuation has been shown to relate with both inflammation and remodelling features [19]. 

In COPD, *in vivo *invasive measurements and *ex vivo *studies of airway resistance have revealed that distal airways are the main site of airflow obstruction in COPD. Using CT, the wall of the small airways <1 mm diameter is beyond the spatial resolution of the technique and cannot be visualized. However large and intermediate airways are not free of abnormalities [30]. The WA indices are increased in COPD smokers, and these dimensions are larger than in smokers without COPD or nonsmokers [31]. Moreover, it has been shown that the mean dimensions of airways with an internal perimeter greater than 7.5 mm are predictive of the mean dimensions of small airways with an internal diameter of 1.25 mm [32]. In COPD patients, correlations were found between WA and LA indices measured by CT with lung obstructive indices measured with PFT, such as FEV1 predicted, forced vital capacity (FVC), residual volume/total lung capacity (RV/TLC), and FEF25%–75% [33] as well as with DLCO [34]. The bronchial wall attenuation measurement has also been assessed in COPD patients. Using the FWHM algorithm, the peak wall attenuation of the bronchial wall was found to correlate with airway obstruction assessed by PFT [17, 18]. 

To the best of our knowledge, few studies have directly compared airway wall remodelling using CT between asthma and COPD, and results are not the same. Data around WA indices indicate either no difference [12, 13], or a larger airway wall thickness in asthmatic patients, thought to be consistent with an increased smooth muscle mass [35]. These apparently conflicting results seem to underly the fact that results around WA indices and correlation with PFTs are not free from variability. This heterogeneity can be partially explained by the lack of consensus regarding patient characteristics and method of WA% quantification. Taken together, studied populations were heterogeneous from one study to another, and either manual or automatic methods of quantification have been used. Regarding the known heterogeneity of bronchial involvement in airway diseases, it could appear critical to know whether a random selection of bronchi [12, 13], a single bronchi measurement as reported on RB1 [15], or an exhaustive quantification of them over the whole bronchi tree is needed to match perfectly the disease reality. Correlations were reported with PFTs, but no pathologic data have been performed to assess any correlation with structural changes in that comparison purpose. Since WA% is aspecific from airway wall components, the smooth muscle mass consistency in asthma [1, 4] could theoretically be balanced with the peribronchial fibrosis displayed in COPD [3]. Hence it remains unclear whether CT can help discriminate between asthma or COPD conditions using WA% quantification, and further assessment is still to be performed to allow it, or not, as a robust standard for a clinical routine practice.

## 3. Quantitative CT of Small Airway

### 3.1. Methods of Quantitative CT Applied to Small Airways

#### 3.1.1. Quantification of Emphysema

Small conductive and distal airways are beyond the spatial resolution of CT in humans. However, the lung parenchyma density, measured on CT scans in Hounsfield Units (HU), results from the X-ray attenuation by intralobular structures, such as alveolar membranes, interstitium, capillaries, or small conductive airways. Therefore, any change in either of them is able to modify the lung attenuation values, and this provides an indirect tool to assess distal airway remodelling. Emphysema is defined as areas of alveolar membrane destruction and loss of the lung elastic recoil around the small bronchi and is a component of airway remodelling seen in severe COPD. Two quantitative methods have been developed [36, 37]. The density mask method consists in applying a density threshold within the lung field to count low-density lung voxels. Then, the density mask technique is defined as the percentage LAA% of total lung volume that contains voxels of lower attenuation than a predefined threshold, usually −950 HU [36]. The percentile method [37] is based on predefined percentages (1%, 5%, or 15%) at which voxels have lower attenuation values. Semiautomatic softwares are available to extract areas of contiguous voxels containing the same lung attenuation values and allow fast and accurate quantification of areas thought to represent emphysematous changes. 

Nevertheless, limitations have been reported. A paradoxical fall in lung density has been reported after smoking cessation, mimicking rapid progression of emphysema in COPD smokers [38]. In that case the lower lung attenuation values have been addressed to an anti-inflammatory effect of smoking cessation which is not to be misinterpreted. Age and lung volume involve density variations too, but not sex gender [39]. Interestingly, attenuation values are modified after contrast application. Therefore, nonenhanced CT scan is to be the reference [40].

**Figure 3:**
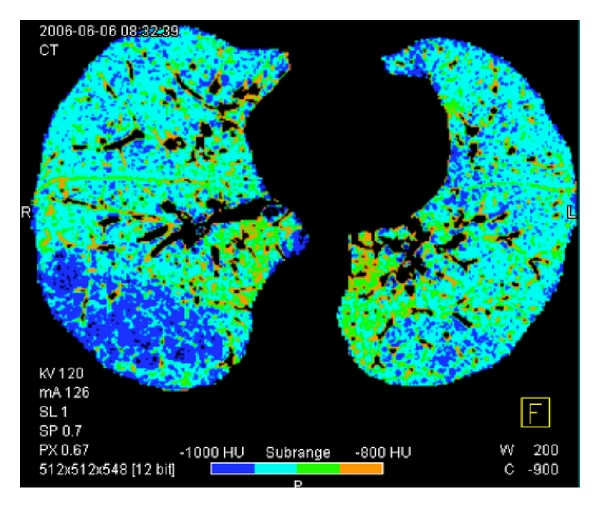
Segmented axial thin section image of right and left lung, from which the mediastinum and lung contours were removed. Using the density technique, dark blue areas indicate voxels between −1000 HU and −950 HU.

Semiautomatic softwares are available to extract areas of contiguous voxels containing the same lung attenuation values and allow fast and accurate quantification of areas thought to represent emphysematous changes (Figure 3).

#### 3.1.2. Quantification of Small Airway Obstruction

Airflow limitation induced by small airway obstruction is defined as the air-trapping phenomenon. Physiologically, air trapping is an abnormal retention of air in the lungs observed in obstructive lung diseases after expiration. The result is an elevated residual volume seen on PFT. On end-expiratory CT scans, the retention of air involves areas of low lung density compared with normal areas of higher value. Nevertheless, low-density areas are not specific for air trapping since emphysema and local hypoperfusion of lung tissue may have the same effect. Various methods have been reported for air-trapping measurement using CT, from visual grading [41] to manual counting [42]. A Semiautomatic method of quantification has been reported using the density mask technique. CT scans performed at forced residual capacity (FRC) showed that the intralobar density value has a mean of −856 HU. Hence, a voxel index of −850 UH on end-expiratory scans has been thought to represent air trapping, whether the lung volume involved is more than 9.66% of the total lung volume [43]. However, there is today no consensus about which one is the best method suitable to be used, and the delineation of a threshold as a cut-off value between trappers and nontrappers is still in process.

#### 3.1.3. Small Airway Remodelling in Asthma and COPD Using Quantitative CT

In asthma, CT lung parenchyma changes have been reported. Laurent et al. [41] showed that the mosaic perfusion pattern was significantly increased at full inspiration in asthmatic subjects, and that this result was addressed to either hypoxic vasoconstriction or small airway obstruction. They also found that the air trapping phenotype was increased in asthmatic and healthy smokers, but not in controls. In asthmatic patients, air-trapping scores correlated with small airway obstruction, assessed by FEV1 and FEF25–75%, which is consistent with small distal airway alterations. Moreover, the focal and diffuse air trapping (E/I ratio) have been shown to correlate with airway wall thickness (WA%) [42]. This should indicate that the remodelling process involves both proximal and distal airways in asthma. 

In COPD, correlations between the extent of emphysema and pulmonary function tests have been well established. LAA% has been shown to correlate with FVC% predicted, FEV1% predicted, FEV1/FVC, RV/TLC, and DLCO/VA [44–50]. However, the major interest of CT should not be to replace PFTs, but to allow quantification of structural modifications and disease phenotypes based on CT imaging. A distribution predominantly in the lower lobe zones has been shown to correlate with obstructive dysfunction and DLCO [51]. Moreover, LAA% has been shown to be a predictor of lung function decline in smokers with normal PFT [52–54], and correlations were found with clinical outcomes [55–57]. A multivariate analysis comparing age, PFT, BMI, and emphysema assessed by CT revealed that LAA% had the strongest association with mortality [58]. 

However, a large amount of overlap exists in clinical practice between asthma and COPD [1, 2]. In a multivariate analysis, Busacker et al. [43] studied a series of asthmatic patients displaying the air-trapping phenotype on their end-expiratory CT scans. The results showed that these patients are more likely to have a history of pneumonia, neutrophilic inflammation, and atopy. Since neutrophilic inflammation is consistent with a Th1-mediated immune response, and not a Th2-mediated as usually seen in asthma, this result should partly explain why an overlap can exist in a clinical practice. Patients are indeed prone to develop *in vivo *both immune responses when they are exposed to various exogenous agents. 

Moreover, both obstruction of the small conductive airways and loss of alveolar attachments are related with destabilisation and premature airway closure during expiration. Therefore, it could be difficult to differentiate emphysema, usually not seen in asthma, from air trapping on CT scans, which can be seen in both diseases. Nevertheless, Matsuoka et al. [59] have reported a quantitative method in order to try differentiating between these two disease conditions. In COPD patients displaying emphysema, they excluded voxels below −950 HU as a validated surrogate of emphysema areas on CT scans. After this first step, they showed that the relative change in lung density between inspiratory and expiratory CT scan had a strong correlation with FEV1, FEF25%–75%, and RV/TLC, using a threshold measured at −860 HU. They concluded that their method is helpful to discriminate air trapping from emphysema in COPD patients.

## 4. Perspectives

CT is of major interest when assessing asthma and COPD *in vivo*. Data coming from histological studies have shown that these two diseases are different regarding their clinical, but also morphological and structural features. However, CT is a monocontrast technique, and there is a lack of specificity, which needs to be addressed. Wall thickness or nonenhanced wall attenuation doesnot allow discriminating between the various airway wall components, and lung attenuation may be altered by any change in intralobular structures. Hence the reported histological differences between asthma and COPD are hard to be transposed to CT. 

To allow a better understanding and comparison of these two diseases using CT, spatial resolution should be improved. Blurring effect alters CT wall thickness quantification of small-to-intermediate airways inferior to 1 mm. This is relevant, knowing the fact that small airways are the main site of disease in COPD. 

Wall density is a new biomarker in asthma and COPD, which attempts to reflect the wall composition instead of the disease extension given by wall thickness. However, no data exists about CT-enhanced wall attenuation value changes using a contrast medium. For instance a hypothetical peribronchial fibrosis enhancement in COPD or chronic asthma should be expected. Specific contrast medium, targeting peribronchial smooth muscle or fibrosis, should be developed. 

MRI techniques using noble hyperpolarized gases have been reported and provide good correlation with CT quantifications. It allows high quality images of ventilation and provides functional information on ventilation cartography or gas diffusion. However, they are cost effective, and their use is still confidential.

## 5. Concluding Remarks

By measuring changes in airway wall and the amount of air trapping, CT may help clarifying the complex physiopathology underlying asthma and COPD and evaluating the effect of treatments. However, CT-derived indices are nonspecific since both inflammation and remodelling may lead to similar changes. Then clinical status and previous use of treatments should be known when interpreting CT changes in these patients. Further developments are suitable; spatial resolution should be improved to prevent blurring of the wall contours; a better delineation of the airway wall contours is needed. Finally the multiplicity of factors of variability in CT measurements should lead to rigorous methods of CT technique. Therefore, the use of quantitative CT is today a research tool and not a daily routine test.

## Abbreviations

COPD: Chronic obstructive pulmonary disease
CT: Computed tomography
LA: Lumen area
WT: Total bronchial area
WA: Wall area
D: External bronchial diameter
L: Internal bronchial diameter
T: Wall thickness
BT: Bronchial wall thickness
HU: Hounsfield Unit
FWHM: Full width at half maximum
PBA: Peribronchial attenuation
PWA: Peak wall attenuation
AHR: Airway hyperreactivity
PFT: Pulmonary function test
FEV1: Forced expiratory volume in one second
FVC: Forced vital capacity
FRC: Functional residual capacity
RV: Residual volume
TLC: Total lung volume
FEF25%–75%: Forced expiratory flow
DLCO: Diffusing lung capacity of carbon monoxide
VA: Alveolar volume
MRI: Magnetic resonance imaging
BMI: Body mass index.
